# The Effects of Anthropogenic Disturbances on the Spatiotemporal Patterns of Medium–Large Mammals in Tropical Volcanic Landscapes

**DOI:** 10.3390/ani13203217

**Published:** 2023-10-14

**Authors:** Nurpana Sulaksono, Satyawan Pudyatmoko, Sumardi Sumardi, Wahyu Wardhana, Arief Budiman

**Affiliations:** 1 Gunung Merbabu National Park, The Ministry of Environment and Forestry, Boyolali 57316, Indonesia; 2 Faculty of Forestry, Universitas Gadjah Mada, Sleman 55281, Indonesia; sumardibdh@yahoo.com (S.S.); wwardhana@ugm.ac.id (W.W.); ariefyogya@gmail.com (A.B.)

**Keywords:** species interaction factor, camera trap, occupancy model, kernel density, Merapi, Indonesia

## Abstract

**Simple Summary:**

Human disturbances affect the activity patterns of mammals. This should be considered when managing national parks, especially in densely populated tropical volcanic landscapes. This study was conducted at the Gunung Merapi National Park between 2019 and 2021, and it aimed to determine the interaction between humans and mammals, both temporally and spatially. We found that medium-sized mammals responded to human activities in complex ways: barking deer (*Muntiacus muntjac*) and leopard cats (*Prionailurus bengalensis*) modified their activity times to avoid encounters with humans, while the wild boars (*Sus scrofa*) tended to avoid humans, choosing locations where there was no human presence. Nocturnal animals tended not to be disturbed because they occupied a different temporal niche to humans. No significant behavioral changes in mammals were observed in response to the human disturbances in this area. However, the presence of poachers and exotic predators (such as domestic dogs) should be a concern to national park managers. As a mitigation effort, human activity should be regulated, both temporally and spatially, in the GMNP. Moreover, education regarding domestic dog ownership and an inventory of dog ownership in the surrounding communities should also be carried out to prevent further disturbances to the mammals in the Merapi volcanic ecosystem.

**Abstract:**

A comprehensive understanding of the consequences of human interactions with mammals is a critical factor in supporting and conserving species in landscapes dominated by humans, which are increasingly threatened. This study aimed to identify the spatial and temporal interactions between humans and mammals. A non-parametric statistical approach with kernel density was used to detect human–mammal temporal interactions. The species interaction factor (SIF) was applied to calculate the spatial overlap based on the two-species occupancy detection model. The activity patterns of medium mammals were nocturnal, diurnal, and cathemeral. The human–medium mammal pairs with SIF values that were <1 and statistically significant included the human–long-tailed macaque (*Macaca fascicularis*) pair, the human–leopard cat (*Prionailurus bengalensis*) pair, and the human–barking deer (*Muntiacus muntjac*) pair. Based on their SIF values and the high overlap in their activity times, the human–macaque pairings had a high risk of conflict. Barking deer and leopard cats displayed a coexistence with humans via time-sharing activities. Due to temporal niche variations with human activities, the existence of nocturnal mammals was relatively uninterrupted. This study showed that most mammals are able to adapt spatially and temporally to various human activities. Nonetheless, efforts to mitigate human–wildlife conflict must be maintained, particularly in the case of severely endangered species, such as the Sunda pangolin.

## 1. Introduction

Tropical mountain forests are habitats for diverse species of endemic flora and fauna [1]. Despite constituting only a quarter of Earth’s total landmass, mountainous areas play a pivotal role as habitats for 85% of global amphibian, mammal, and avian populations [2]. This circumstance designates these areas as biodiversity hotspots, underscoring the imperative to conserve and safeguard these natural habitats [3]. Currently, mountain forests are at risk from escalating human activity. The existence of humans in forest areas is inevitable and unavoidable [4,5]; the high level of human interaction with forest areas makes it difficult to eliminate human presence, which continues to have an impact on wildlife [6]. This is especially true in human-dominated landscapes, in which the easy access to natural forests means that humans are closer to wildlife [7]. Human activities can cause alterations in animal behavior [8,9,10] and, globally, they even contribute to the extinction of numerous mammalian species [11,12]. Wildlife populations are in decline due to increased human activity, which promotes habitat fragmentation and degradation.

Human–wildlife interaction (HWI) is defined as the juxtaposition of human and wildlife activity in space and time, which influences humans or wildlife, or both [12,13]. HWI can be positive, neutral, or negative [13]. Positive interaction occurs when animals benefit from humans, such as hikers who provide food for small mammals [14] or tourists who protect animals from predator attacks and provide pathways for animals [15]. However, human–wildlife interactions may lead to negative consequences and are commonly referred to as human–wildlife conflicts. These interactions have the potential to inflict harm on both humans and animals. Multiple studies have documented incidences of human conflict with large mammals [16,17,18], primates [19,20], and even with medium-sized mammals [21]. The issue of human–wildlife conflict is a widespread global challenge that continues to necessitate the exploration of viable resolutions.

Conflicts occur due to the high level of spatial and temporal human–wildlife interaction in a given landscape [22,23,24], as humans and wildlife must compete for resources and space [25]. Consequently, wild animals respond to human disturbances by diversifying via behavioral changes or by migration/evasion [10]. Previous research has discussed, among other things, the following responses: changes in the behavior of *Bos javanicus* due to human disturbances in the Baluran National Park [26]; changes in deer activity due to exotic predators [26]; changes in bird behavior on weekends to avoid tourist activities [27]; changes in animal activities due to hunting [27,28,29]; and habitat fragmentation due to settlements, plantations, and roads [9]. Human activity can interfere with animals’ sustenance and companionship [30,31,32]. Changes in behavior also occur when the availability of resources is no longer sufficient to support them [33], causing animals to seek out alternative sources. The evasion method can be undertaken temporally, by altering the time of activity [34], or spatially, by migrating to an area that is safe and has sufficient resources. These spatiotemporal activity patterns are a form of adaptation that enhances animals’ resiliency and decreases their susceptibility to extinction [35]. Conflicts between humans and wildlife have negative consequences for both.

Understanding the variety in HWIs is essential to improving our understanding of how complex the causes of these conflicts are [13,22,23]. Coexistence is a dynamic state in which humans and wildlife share space in the same landscape [36]. Consequently, identifying the patterns in human–wildlife interactions is a fascinating tool for the development of future mitigation measures to foster human coexistence with wildlife. While previous research on the patterns of HWIs has been extensive [37,38,39,40,41,42], research regarding active volcanic mountain landscapes that are surrounded by human settlements are rare.

The Gunung Merapi National Park (GMNP), a tropical mountain forest, is a habitat for species of mammals that are threatened by human disturbances [43]. It is located on Java Island, which is on the border between the provinces of central Java and Yogyakarta. The park is one of the most active volcanoes in Indonesia and it is surrounded by densely populated settlements. Eruptions occurred more than 25 times between 1900 and 2010. The largest eruption (measuring four on the Volcanic Explosivity Index (VEI = 4)) was in 2010. It caused significant damage, killing, injuring, and displacing between 367,277 and 410,388 people, and causing damage to an estimated 2300 homes [44]. This volcanic event also affected the biological community, with long-term implications [45].

The GMNP is an open ecosystem, so it is accessible by humans. After the significant volcanic eruption in 2010, the local people resumed their traditional use of the natural resources inside the national park. They have been interacting with Mount Merapi’s forest, the habitat of many mammals, for a long time, even before the forest was declared a national park. This issue is prevalent in protected areas that are immediately adjacent to human settlements, as well as those that are close to agricultural or plantation areas [46,47]. Therefore, the use of mitigation measures is necessary to reduce the adverse effects of humans on the environment [48], and understanding the impact of disturbances on animals is critical for developing this mitigation planning.

This study aimed to investigate the influence of human disturbances on medium-sized mammals within the landscape of the GMNP. The objectives were presented as three specific goals: (1) to measure the spatial interaction between humans and large mammals, (2) to measure the temporal interaction between humans and medium-sized mammals, and (3) to determine the medium-sized mammals’ responses to the presence of humans. Theoretically, the creatures that inhabit landscapes that are dominated by humans alter their behavior spatially and/or temporally [49,50]. We hypothesized that medium-sized mammals would adapt to human activity by adjusting their activity times and spaces [49,51]. Wild animals will negotiate the existence of humans in space and/or time to avoid direct encounters with humans. Some mammals have a tendency to adapt so that they can coexist with humans, and sensitive mammals tend to avoid humans and select a different habitat. The results of this research could be useful for measuring the level of human disturbance and for identifying the risks these disturbances pose to wildlife. Changes in animal behavior and activity can serve as indicators of ecosystem disturbances.

## 2. Materials and Methods

### 2.1. Study Area

**Figure 1:**
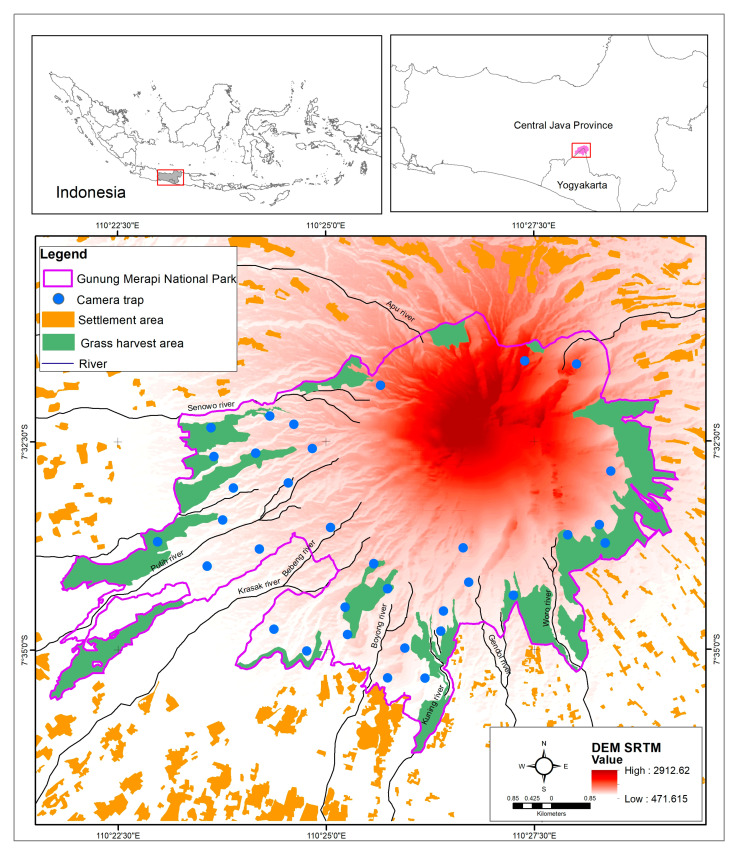
The geographical location of the Gunung Merapi National Park. The yellow area is the settlement that surrounds the GMNP. The green area is the location of the grass harvest. The dark red area is the summit of Mount Merapi. The placement of camera traps (blue circles) adjusted the shape of the study area withing the national park so that most of it was on the southern and western slopes, and so it avoided areas prone to pyroclastic flows, which were in a radius of 3 km from the summit. (Source: Gunung Merapi National Park Office, 2019; Digital Elevation Model of Indonesia and Indonesia Topographical Map were accessed from https://tanahair.indonesia.go.id/portal-web/ (accessed on 22 July 2021)).

This study was conducted within the confines of the Gunung Merapi National Park, a volcanic terrain with an elevation of 2930 m above sea level. The location is positioned on the border between the provinces of central Java and Yogyakarta (7°35′ S and 110°24′ E). Furthermore, the park has a total expanse of 6442 hectares (Figure 1), and it is amidst an assemblage of 30 surrounding villages. The population density measured 3627 individuals per km^2^, and the villages are situated at altitudes ranging from 505 to 1629 m asl.

The topography of the national park exhibits an inclination towards the south-west, which was the most impacted area when Merapi erupted; therefore, local communities use its fertile land for agricultural activities [52,53]. A total of 21.1% (1358 ha) of the national park’s area is used for grass harvesting to feed livestock and seven of the 13 rivers are sand mining sites [54].

### 2.2. Data Collection

Passive infrared digital camera traps were used to acquire spatial and temporal data about the presence of mammals. A total of 17 cameras, consisting of 16 Bushnell Trophy cams (HD 1119836c) and one Bushnell Trophy cam (HD 119537c model), were systematically rotated and sequentially stationed across 34 sites between July 2020 and February 2021. Each station served as a discrete sampling unit, covering an area of 1 km^2^. To ensure the collection of geographically distinct presence detection data, the cameras were positioned at an average interval of 1.1 km, with an operational duration average of 71.35 days per camera. The strategic placement was orchestrated to optimize animal identification within each sample unit, accounting for factors such as trail crossings among the ridges [55,56]. A challenge arose because of the area’s topographical configuration, which featured two to three ridges interspersed by precipitous inclines. The cameras were affixed approximately 20 to 50 cm above ground level at their respective locations and no bait was used to entice the animals. The imagery was taken at 2-s intervals between exposures, capturing a triad of photographic sequences for each trigger event. The cumulative tally of camera days used for sampling amounted to 2426 days. Each image contained the timestamp and date, a feature facilitated by the configuration. A Garmin 78s device was used to document the geographical coordinates of each position.

The presence of species in a specific location was determined by the interaction between species, such as humans and animals, but also by the characteristics of the habitat [57,58]. The attributes recorded at each camera location were (1) the density of vegetation cover (HDF); (2) the slope (SLO); (3) the slope or ridge aspect (ASP); (4) the Euclidean distance to the nearest settlement (DFS); (5) the Euclidean distance to the sand mining area (DFM); (6) the Euclidean distance to the lava dome area (DFD); (7) the Euclidean distance to the grass harvest area (DFG); (8) the altitude of the location (HIG); and (9) the Euclidean distance to the mountain summit (DFP) (Table S1).

The collection of vegetation data involved the establishment of 170 plots inside the area observed by the camera traps. These plots were placed in radial patterns, with a radius of 100 m. There was one plot at the location of each camera trap and an additional four were positioned in the cardinal directions. Furthermore, the measurement of the vegetation density was conducted by calculating the average value of the five plots per camera site, focusing on the variable canopy cover. The slope and aspect values were computed using the SAGA (System for Automated Geoscientific Analyses) 8.1.0 software, with the Digital Elevation Model (DEM) data obtained from Google Earth Engine [59,60].

### 2.3. Data Analysis

The tagging of individuals and species of mammals in each photograph was performed using the open-source software digiKam 7.1.0 [61,62], which may be accessed at https://www.digikam.org (accessed on 22 March 2021). Further data processing was carried out using the open-source metafile management–organization workflow R package camtrapR version 2.0.3 and R version 4.1.1 [62,63], which resulted in a compilation of survey data. The data collection containing the image metadata (for example, the species, location, time, and hour) was then utilized to prepare a detection–occupancy analysis in the form of creating historical detection data for each species [62,64].

### 2.4. Spatial Interaction Analysis

The conditional two-species occupancy model, with the covariates used by Richmond et al. (2010), was used to examine the effects of habitat quality and human activity on the distribution and occupancy of mammal species. The presence of the dominant species determined the probability of the occupancy of subordinate species in this occupancy model [65,66]. This model was used to assess variations in the likelihood of detecting a specific species based on the co-presence of one or both species. Furthermore, it was used to explore when the detection of a subordinate species was contingent upon the identification of the dominant species in cases of concurrent presence. The parameterization between human and mammal species in this study was as follows. The occupancy probability for species A (the dominant species, humans) was unconditional, whereas the occupancy probability for species B (the subordinate species, mammals) was conditional, depending on the presence or absence of species A [40].

The conditional likelihood-based, two-species occupancy model generated nine parameter estimates [65,67,68], four of which were important and used in the analysis (Table S2). The four parameters were the occupancy probability of species A (ψA); the occupancy probability of species B when species A was absent (ψBa); the occupancy probability of species B when species A was present (ψBA); and ɸ, the spatial interaction factor of the two species (SIF). An SIF greater than one indicated that the two species occur together more frequently than would be expected by chance. An SIF less than one showed that the two species occur together less frequently than would be expected by chance. SIF = 1 implies that the two species occur independently (no aggregation or avoidance) [68].

Using a single-season, single-species occupancy model, the probability of the detection (p) and occupancy (ψ = the percentage of space used) of mammals were evaluated in the two-species occupancy model [58]. The detection history was used to estimate the occupancy probabilities by adding environmental data (for example, habitat attributes as site covariates) to the model to determine the effects of habitat variables on the species detection probabilities and occupancy. The maximum likelihood estimation was used to estimate important covariates for the occupancy probability model and detection using the likelihood function [58]. The corrected Akaike information criterion (AICc) was used to rank the models and evaluate their strengths by comparing the AICc scores, model weight (wgt), and deviation differences (−2log probability) [69].

The model was selected when the ∆AICc was less than two and the weight > 0.1 [70,71]. The model averaging method was used to weigh and select the significant environmental factors for each occupancy probability model. Variables with a weight less than 0.15 were omitted [72]. The occupancy model was analyzed using the unmarked R package [73,74]. The relative importance of the parameters were computed using the R package AICmodavg [72]. The best model was used to calculate four parameters in the two-species occupancy model [67]. The top model was imported into the PRESENCE Program application, version 2.12.9, for the analysis of the single-season, two-species occupancy model. This was conducted using a choice of co-occurrence ψBa/pBa parameterization, which generated estimates for ψA, ψBA, ψBa, and the SIF [65,68].

### 2.5. Temporal Interaction Analysis

The investigation of activity patterns and the daily overlap of 24-h cycles between species A and B was conducted through a three-step process [75,76], using camera trap data. First, we determined the overlapping coefficient, ∆, which ranged from zero (no overlap) to one (complete overlap) [77]. The analysis was conducted using the overlap package in the R programming language [56]. A technique was used to modify the kernel density estimation function based on the duration of the animal observations (referred to as animal active time) captured by the camera trap. Second, we determined the estimator based on the size of the smaller of the two samples. The smaller sample was less than 75, the estimator ∆1 performed best, and ∆4 was better when it was greater than 75 [77]. We used estimator ∆1, adjusted to 0.8 due to the amount of data < 50. Third, we determined a confidence interval (CI) of 95% with 10,000 bootstraps [63,77]. The analysis involved generating kernel density curves and fitting and quantifying the degree of overlap. The Rayleigh test was carried out on the circular data for each species using the circular package in R to evaluate the time activity data [78]. The Mardia–Watson–Wheeler test was performed to assess the statistical significance of the differences between the two activity patterns [79]. The null hypothesis pertaining to the common distribution was deemed invalid when the value of the Mardia–Watson–Wheeler test exceeded the critical value, as denoted by a significance level of *p* < 0.005 [80].

## 3. Results

A total of 1370 independent photographs were captured during the study (Table 1), consisting of 471 long-tailed macaques (*Macaca fascicularis*); 264 barking deer (*Muntiacus muntjac*); 156 Sunda porcupines (*Hystrix javanica*); 142 Asian palm civets (*Paradoxurus hermaphroditus*); 77 leopard cats (*Prionailurus bengalensis*); 54 Javan ferret badgers (*Melogale orientalis*); 54 small Indian civets (*Vivirricula indica*); 30 wild boars (*Sus scrofa*); and 16 Malayan pangolins (*Manis javanica*). The three species with the lowest number of encounters were the Javan mongoose (*Herpestes javanicus*), the Javan langur (*Trachypithecus auratus*), and the giant red flying squirrel (*Petaurista petaurista*), with 11, five, and six encounters, respectively. These three species were not analyzed further because they had a small sample size [81,82]. The camera trap captured 57 images of human activity, consisting of three at night and 54 during the day, such as collecting grass or firewood, driving two-wheeled vehicles, poaching, and conducting field research. Additionally, it also captured images of dogs, birds, and small mammals, such as tree shrews (*Tupaia glis*), the Javan tree shrew (*Tupaia javanica*), the plantain squirrel (*Callosciurrus notatus*), the three-striped ground squirrel (*Lariscus insignis*), and mice.

The species with the highest occupancy were the long-tailed macaque (0.94 ± 0.04), the Asian palm civet (0.80 ± 0.07), and the barking deer (0.65 ± 0.09). However, the Malayan pangolin, with a value of 0.22 ± 0.18, had the lowest occupancy probability (Table 1). Other mammals with a similar probability were the Javan ferret badger (0.48 ± 0.13), the Sunda porcupine (0.44 ± 0.09), the leopard cat (0.59 ± 0.09), the small Indian civet (0.36 ± 0.11), and the wild boar (0.33 ± 0.12).

### 3.1. Spatial Interaction

The mammalian species with a high probability of occupation (ψBA > 0.5) when humans were present were dogs (0.76), the barking deer (0.85) (Table 2), the long-tailed macaque (1.00), the leopard cat (0.94), and the small Indian civet (0.61). The mammalian species with a high probability of occupation (ψBa > 0.5) when humans were absent were the Malayan pangolin, the Asian palm civet, the long-tailed macaque, and the barking deer. Three mammal species had high ψBA and ψBa values: the long-tailed macaque, the barking deer, and the Asian palm civet.

The pairs that had a negative spatial interaction, with an SIF value < 1, and that were statistically significant (i.e., 95% CI did not overlap one) were the human–Malayan pangolin pair (SIF Value 0.24; CI 0.19–0.45); the human–wild boar pair (SIF value 0.33; CI 0.03–0.63); and the human–Sunda porcupine pair (SIF value 0.6; CI 0.23–0.98). The mammals in these pairs had a low probability of co-occurring with humans randomly. Furthermore, five pairs had SIF values ≥ 1: the human–dog pair (SIF value 1.55; CI 0.56–2.53); the human–leopard cat pair (SIF value 1.21; CI 0.93–1.49); the human–small Indian civet pair (SIF value 1.23; CI 0.66–1.81); the human–barking deer pair (SIF value 1.19; CI 0.90–1.48); and the human–long-tailed macaque pair (SIF value 1.06; CI 0.97–1.15).

### 3.2. Activity Patterns and Temporal Interaction

According to Table 3, there were three activity patterns for medium mammals in the Mount Merapi ecosystem: nocturnal, diurnal, and cathemeral. The Sunda porcupine, Malayan pangolin, Javan ferret badger, Asian palm civet, small Indian civet, and the giant red flying squirrel were the animals with nocturnal activity patterns. These six animal species were mostly nocturnal, with over 90% of their activity being recorded at night. The wild boar, Javan mongoose, long tailed macaque, and the barking deer were diurnal animals. The animals with more than 85% of their activities recorded in the daytime were the Sunda porcupine, Javan langur, wild boar, and the long-tailed macaque. The leopard cat is a cathemeral mammal: it was sometimes active during the day (39% of the time) and sometimes active at night (61% of the time). The majority of human activities were seen to occur during the day, between 07:00 and 14:00, with peak activity between 09:00 and 12:00. However, human activity was also seen late at night, with three (5%) of the 57 independent photographs depicting human activity at night. Furthermore, domestic dog activity was recorded during the day (74%) and at night (26%). The Rayleigh uniformity test results showed that the temporal activity of medium-sized mammals followed specific patterns for each species (Table 4).

**Figure 2:**
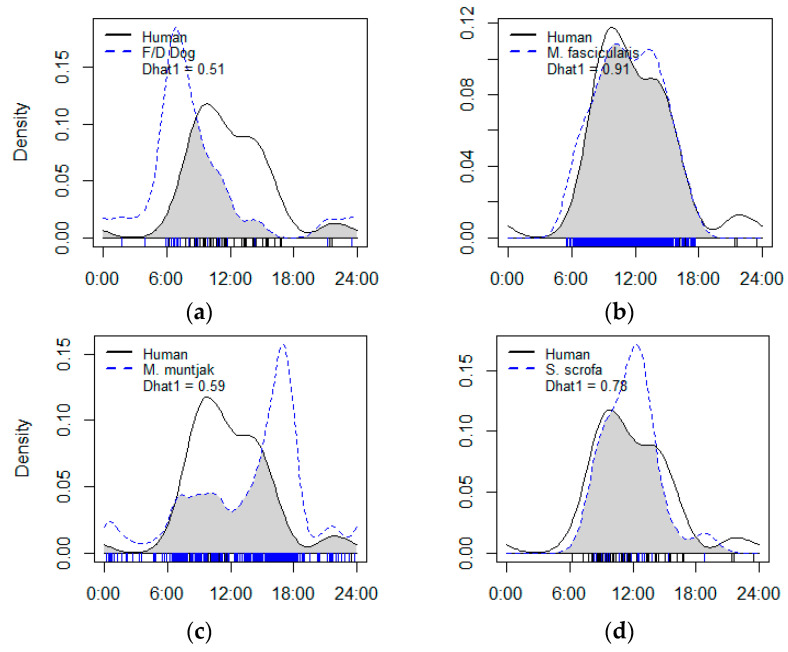

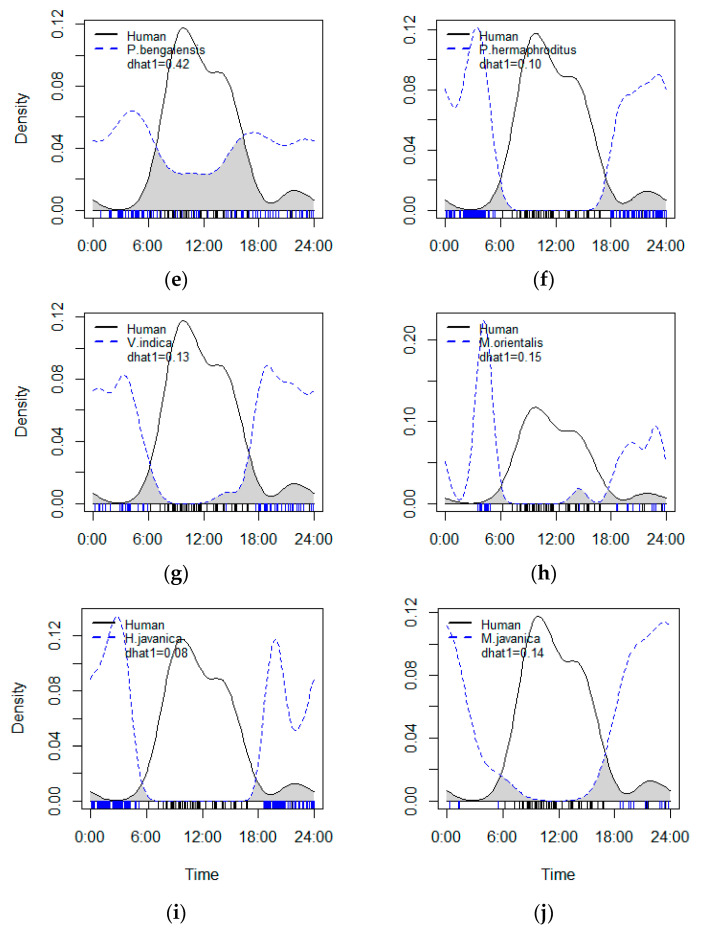
Overlap in the daily activity patterns between the following pairs: (**a**) human–dog, (**b**) human–*M. fascicularis*, (**c**) human–*M. muntjac*, (**d**) human–*S.scrofa*, (**e**) human–*P.bengalensis*, (**f**) human–*P.hermaphroditus*, (**g**) human–*V.indica*, (**h**) human–*M. orientalis*, (**i**) human–*H. javanica*, and (**j**) human–*M. javanica*.

The results of the Mardia–Watson–Wheeler test revealed that the daily activity patterns of all the human–mammal pairs were significantly different (*p* < 0.05) (Table 5). The long-tailed macaque, wild boar, and barking deer had similar daily activity patterns to humans. However, the activity of the long-tailed macaque and humans had the highest overlap (0.91), with a 95% confidence interval of 0.83–0.98. The peak activity time of the long-tailed macaque was also nearly identical to humans, at between 9:00–12:00. This species also showed a nearly identical activity pattern, with an overlap value of 0.78 and a 95% confidence interval, ranging from 0.59–0.91. However, the wild boar species showed peak activity between 07:00 and 10:00, which differed from humans. The degree of temporal overlap between the activities of the barking deer and humans was estimated to be 0.59, with a 95% confidence interval between 0.48–0.68. The peak activity time of the barking deer also differed, occurring between 15:00 and 18:00. Figure 2 shows that the activity of the barking deer increased when human activity began to decline, between 14:00 and 18:00. The nocturnal species were seen to have a low degree of temporal overlap in their activity patterns with human activities. This was a result of the time difference between humans and nocturnal animals. The Sunda porcupine had the lowest overlap value at 0.08, followed by the Asian palm civet, the small Indian civet, the Malayan pangolin, and the Javan ferret badger, with values of 0.10, 0.13, 0.14, and 0.15, respectively. In addition, the peak time for nocturnal animal activity also differed from that of human activity. Human activity peaked during the day, whereas the nocturnal species activity peaked at night; therefore, the time overlap between humans and nocturnal animals was low (Figure 2).

## 4. Discussion

Human disturbances have direct and indirect effects on animal activities [8,9], such as changes in behavior regarding foraging for food, finding mates, altering activity patterns, and avoiding predators. Animals respond in several ways to human disturbances, including avoidance, behavior modification, and coexistence. Animals sometimes opt to remain in proximity to the human presence as a means of safeguarding themselves from predator attacks and ensuring a source of sustenance [15,38]. This study provides ecological answers to issues about the relationship between humans and medium-sized mammals in the world’s most active volcanic and residential areas.

Our study found patterns of both low and significant spatial interaction (SIF < 1) between humans and nocturnal species, specifically the Malayan pangolin. The IUCN Red List classifies this mammal as a critically endangered species, whose population in its native habitat is beginning to be threatened with extinction [83]. The Malayan pangolin is a favored target for illegal hunters, who pose a significant threat to the species due to its excessive exploitation for both domestic and global purposes [84]. We identified that the Malayan pangolin is a nocturnal animal, which was consistent with previous studies conducted in Ujung Kulon National Park [85]. It has a low and significant spatial interaction with humans, as well as an overlap time value regarding its activity (i.e., 0.20). The Malayan pangolin is a species of mammal that avoids human interaction. This research supports the results of a previous study, which showed that the sensitivity of this species to human activity is very high, even though it can adapt to different types of forest cover in plantations or natural forests, provided food is abundant [86]. In the Bukit Barisan National Park, this species has been found to avoid areas that are disturbed by human activity, such as roads and settlements, and threats, such as poaching [87,88]. The interaction between humans and the Malayan pangolin in the GMNP was found to be low, both spatially and temporally. This species has the ability to select a location that is different from human-occupied space. The daily activity pattern of the Malayan pangolin in the GMNP ecosystem has not changed. This study also found that the native occupancy of the Malayan pangolin was low compared to other species. Therefore, conservation efforts are continuously being strengthened to ensure its survival in national parks.

Like the Malayan pangolin, the Sunda porcupine was seen to be nocturnal, with low and significant spatial and temporal interactions with humans. The trap cameras captured a variety of porcupine behaviors, including searching for food, mating, and trail crossing. A total of 156 porcupine images were captured by the camera traps at 14 stations, all at night. This study supported the results of previous studies, that the Sunda porcupine is a nocturnal species [51,82]. We detected no modifications in the temporal pattern of porcupine activity in the GMNP. We recorded an SIF value of less than one, which was statistically significant, showing that the Sunda porcupine tended to stay away from humans. The spatial segregation of the Sunda porcupine may be due to the abundance of food sources in the national park. The Sunda porcupine’s diet is quite diverse, including tubers, fruits, seeds, young shoots, flowers, and other plants [89,90]. Nevertheless, the existence of the Sunda porcupine in the GMNP should be a concern for national park managers. In some areas, a negative interaction is as harmful to humans as to animals, such as crop destruction [91]. The high demand for the Sunda porcupine makes this animal a tempting target for poachers [91]. Therefore, despite its status of ‘least concern’ on the IUCN Red List, this species is protected under Indonesian law [92].

The spatial interaction between the human–Asian palm civet and the human–Javan ferret badger each had an SIF value of less than one. However, it was not significant because the 95% CI value overlapped one. Both species were spatially unaffected by human activities in space utilization because the GMNP has an abundant food source for civets. The Asian palm civet and the Javan ferret badger are both omnivorous, with a dietary preference for forest fruits; seeds; and small mammals, including rodents [93]. This dietary flexibility enables these species to display a higher degree of adaptation to their various environments. Additionally, the temporal interaction of both the Asian palm civet and the Javan ferret badger had minimal values in their time overlap with humans. The difference in the temporal niche occupied by people and these animals in this particular activity contributes to its enhanced safety and to reduced negative effects from human interference/disturbance. The presence of humans does not interfere with these animals’ spatial and temporal behavior.

Wild boars are diurnal animals, which are primarily active during the day. The results of this study were in accordance with previous studies [29,94,95]; however, other studies have also categorized wild boars as cathemeral [85]. The activity times of the wild boars in the GMNP are almost the same as the local community activity times to search for grass and firewood. This was indicated by the high overlap value of wild boars’ activity times with human activity times: 0.78 (Figure 2d). However, this overlap did not interfere with the wild boars’ activity times because of the spatial niche differences. The spatial interaction value between wild boars and humans was low and statistically significant (SIF < 1). Wild boars adjusted spatially by choosing different spaces from humans. The wild boar–human pairs were seen to coexist spatially in the GMNP’s landscape. This condition must be maintained because several studies have found negative interactions that cause changes in wild boars’ activity patterns [34,96]. The distribution of wild boars is extensive, spanning several countries. Due to their abundance, they can become a problem for humans as they frequently raid crops [97].

The long-tailed macaque, leopard cat, and barking deer had high spatial interactions with humans (SIF > 1), although this was not statistically significant. The time overlap between the long-tailed macaque and humans was high (0.91), while the overlap between the leopard cat and barking deer with humans were medium. Human conflict is possible with these three types of animals, especially the long-tailed macaque. Several studies have discussed long-tailed macaque conflicts with humans in the Mount Merapi landscape [98]. However, human conflict with leopard cats and barking deer have not occurred. We estimated that leopard cats and barking deer would adapt temporally to human activity times, but within a narrow time niche. These species adjusted the time of their activity to prevent coinciding with that of the human community’s collection of grass.

Figure 2 shows the difference in the peak activity times of barking deer and humans. The peak activity of barking deer was between 15:00 and 18:00, while that of humans was between 09:00–12:00. These data show that barking deer tended to select a different activity time to humans: the afternoon, before sunset. This is a form of animal adaptation, which helps them to survive changes in their environment. Behavioral changes develop into activity patterns when a disturbance occurs continuously [8,10]. This process has happened to barking deer in the Gunung Merapi National Park area. Afternoons and early evenings were the best times for these animals because human grass collecting declined at these times of the day.

The study also recorded the presence of domestic dogs and that there was a positive relationship with humans (Figure S1), as shown by a SIF value of more than one and ψBA > ψBa. The results of this study strengthened those of previous research [99]. The dogs’ activity was not limited to during the day alone but was observed through the night until early in the day. Local people introduced the first domestic dogs into the landscape. These dogs’ activities occurred between 06:00 and 14.00 when they accompanied their owners into the forest. A dog appeared to be in charge of protecting its owner from wildlife disturbances or other threats. The poachers introduced the second type of dog, and its activity time was the same as that of its owner’s, between 20:00 and 02:00. The dogs were grouped and assigned to chase target animals, such as barking deer, wild boar, and the Malayan pangolin. These results are consistent with the previous findings, which suggested that the presence of dogs in forest areas is due to humans [47]. The existence of human settlements around the conservation area has caused an increase in the population of domestic dogs. Therefore, the existence of domestic dogs should be considered as a serious concern for area managers. Previous studies found that the presence of domestic dogs caused changes in the native mammal species in terms of their reproductive processes, food requirements, and distribution [32,100]. Therefore, future studies should be conducted to detect the interactions between exotic predators and native mammals in the GMNP.

## 5. Conclusions

This research provides new quantitative information about the spatial and temporal interactions between mammals and humans. Based on this study, it is known that medium-sized mammals respond to human activity in a complex way. Some types of mammals choose to modify the times in which they are active to avoid encounters with humans, just as they do with frogs and leopard cats. Wild boars are more likely to choose to adjust their activity spatially by using areas that rarely see human activity. Nocturnal animals (the Javan ferret badger, the Malayan pangolin, and the Sunda porcupine) tend not to be disturbed by human activity because they have different time niches; although, some nocturnal animals have more than one SIF value, such as the Asian palm civet. Based on some of these findings, it is known that, although it has open access, in general, the area of the Gunung Merapi National Park still currently provides space for various types of terrestrial mammals. No significant changes in animal behavior were found, such as changes from diurnal to nocturnal or vice versa, as a response to disturbance. The mammals were still able to adjust both spatially and temporally to various human activities. The continuing volcanic eruptions have brought their own benefits to the mammals in this landscape. The establishment of an eruption hazard area within a 3 km radius from the peak has ensured that the area is free from human activity; therefore, it is a safe place for animals to be active. The existence of illegal hunters and exotic predators should concern national park managers as they are dominant and can cause threats and disturbances to wildlife. Furthermore, we recommend that further research on the relationship between exotic predators and native mammals in the GMNP be conducted to further supplement the current information related to the disorders present in Merapi. As an effort to mitigate the negative effects of human disturbances on mammals, it is necessary to regulate human activity in the GMNP area, both temporally and spatially.

## Figures and Tables

**Table 1:** Single-season occupancy model results of the mammals in the Gunung Merapi National Park.

Model		No. of Independent Captures	Relative Abundance Index	Naïve Occupancy	Probability of Occupancy ψ ± SE	Elevation Mean (Min–Max)
Human	ψ(.)p(.)	57	4.19	0.26	0.27 ± 0.08	1145 (980–1575)
Domestic dog	ψ(.)p(.)	27	1.99	0.29	0.30 ± 0.10	1381 (986–2155)
Barking deer (*Muntiacus muntjac*)	ψ(.)p(.)	264	19.43	0.65	0.65 ± 0.09	1313 (980–2155)
Wild boar (*Sus scrofa*)	ψ(.)p(.)	30	2.21	0.32	0.33 ± 0.12	1320 (991–1867)
Long-tailed macaque (*Macaca fascicularis*)	ψ(.)p(.)	471	34.66	0.94	0.94 ± 0.04	1262 (848–2155)
Leopard cat (*Prionailurus bengalensis*)	ψ(.)p(.)	77	5.67	0.56	0.59 ± 0.09	1313 (980–2155)
Asian palm civet (*Paradoxurus hermaphroditus*)	ψ(.)p(.)	142	10.45	0.79	0.80 ± 0.07	1253 (848–1867)
Small indian civet (*Viverricula indica*)	ψ(.)p(.)	54	3.97	0.35	0.36 ± 0.11	1242 (959–1575)
Javan ferret badger (*Melogale orientalis*)	ψ(.)p(.)	54	3.97	0.47	0.48 ± 0.13	1321 (848–1867)
Sunda porcupine (*Hystrix javanica*)	ψ(.)p(.)	156	11.48	0.41	0.44 ± 0.09	1215 (848–1648)
Malayan pangolin (*Manis javanica*)	ψ(.)p(.)	16	1.18	0.21	0.22 ± 0.18	1319 (1016–1687)

**Table 2:** Estimates of the parameters of conditional co-occurrence of human and subordinate species in the Gunung Merapi National Park. The best single-species occupancy model of the dominant species was used to calculate the model. The parameter estimates with standard errors (SE) included A (the probability occupancy of dominant species); BA (probability occupancy of subordinate species when dominant species A is present); Ba (probability of occupancy of subordinate species when dominant species A is absent); and the species interaction factor (SIF), with a corresponding 95% confidence interval, as well as the lower and upper estimates.

Model		ψA	ψBA	ψBa	Φ (SIF)	φ (95% CI)
Human–domestic dog	ψ(.)p(ASP.DFG.DFM.DFS.HDF.HIG.SLO)	0.60	0.76	0.08	1.55	0.56–2.53
	SE	0.12	0.02	0.01	0.20	
Human–wild boar	ψ(.)p(ASP.DFD.DFM.DFS)	0.38	0.23	0.15	0.33	0.03–0.63
	SE	0.11	0.01	0.02	0.02	
Human–barking deer	ψ(.)p(ASP.DFM.DFS.HDF.LDF)	0.42	0.85	0.61	1.19	0.90–1.48
	SE	0.09	0.10	0.01	0.01	
Human–long-tailed macaque	ψ(.)p(DFM.DFS.HIG.LDF)	0.66	1.00	0.83	1.06	0.97–1.15
	SE	0.09	0.00	0.11	0.05	
Human–leopard cat	ψ(.)p(ASP.DFM.DFS)	0.64	0.94	0.49	1.21	0.93–1.49
	SE	0.09	0.07	0.02	0.01	
Human–Asian palm civet	ψ(.)p(ASP.DFM.DFS.HDF)	0.49	0.88	1.00	0.94	0.84–1.03
	SE	0.09	0.08	0.00	0.05	
Human–small Indian civet	ψ(.)p(ASP.DFD.DFP.DFS)	0.51	0.54	0.33	1.23	0.66–1.81
	SE	0.09	0.01	0.02	0.03	
Human–Javan ferret badger	ψ(.)p(ASP.DFD.DFP.LDF.SLO)	0.60	0.61	0.43	0.80	0.53–1.06
	SE	0.02	0.01	0.03	0.01	
Human–Sunda porcupine	ψ(.)p(ASP.DFG.DFM.DFP.HDF.HIG.LDF)	0.67	0.34	0.28	0.60	0.23–0.98
	SE	0.01	0.02	0.01	0.02	
Human–Malayan pangolin	ψ(.)p(ASP.DFG.DFM.HIG)	0.31	0.10	0.56	0.24	0.19–0.45
	SE	0.09	0.09	0.18	0.03	

**Table 3:** Number of station, number of images, period of encounter, and category of medium-sized mammals in the Gunung Merapi National Park.

Species	English	No. of Stations	No. of Images	No. of Events		Period		Activity Category
				Night	Day	Night	Day	
Human		10	57	3	54	0.05	0.95	-
Domestic dog		9	27	7	20	0.26	0.74	-
*Macaca fascicularis*	Long-tailed macaque	32	471	11	460	0.02	0.98	D
*Herpestes javanicus*	Javan mongoose	9	11	0	11	0.00	1.00	D
*Sus scrofa*	Wild boar	11	30	1	29	0.03	0.97	D
*Muntiacus muntjac*	Barking deer	22	264	53	211	0.20	0.80	D
*Prionailurus bengalensis*	Leopard cat	19	77	47	30	0.61	0.39	C
*Viverricula indica*	Small Indian civet	11	54	50	4	0.93	0.07	N
*Melogale orientalis*	Javan ferret badger	16	54	53	1	0.98	0.02	N
*Paradoxurus hermaphroditus*	Asian palm civet	26	142	141	1	0.99	0.01	N
*Manis javanica*	Malayan pangolin	7	16	16	0	1.00	0.00	N
*Hystrix javanica*	Sunda porcupine	14	156	156	0	1.00	0.00	N
*Trachypithecus auratus*	Javan langur		6	0	6	0.00	1.00	-
*Petaurista petaurista*	Red giant flying squirrel	1	5	5	0	1.00	0.00	-

Note: D—diurnal; N—nocturnal; and C—cathemeral.

**Table 4:** Rayleigh uniformity test for the temporal activity of human and subordinate species in the Gunung Merapi National Park.

Species	N	Rayleigh Test	
		R	*p*
Human	57	0.72	<0.0001
Domestic dog	27	0.74	<0.0001
Wild boar	30	0.89	<0.0001
Barking deer	264	0.58	<0.0001
Long-tailed macaque	471	0.74	<0.0001
Leopard cat	77	0.54	<0.0001
Asian palm civet	142	0.65	<0.0001
Small Indian civet	54	0.50	<0.0001
Javan ferret badger	54	0.53	<0.0001
Sunda porcupine	156	0.72	<0.0001
Malayan pangolin	16	0.84	<0.0001

**Table 5:** Overlap coefficient (Δ1) between the human and medium-sized mammals’ activity patterns, 95% confidence intervals (95% CIs), and Mardia–Watson–Wheeler test (MWW).

	Δ1 (95% CI)	MWW	*p* Value
Human–domestic dog	0.51 (0.41–0.68)	30.27	*p* < 0.001
Human–wild boar	0.78 (0.59–0.91)	3.9788	*p* < 0.001
Human–barking deer	0.59 (0.48–0.68)	47.679	*p* < 0.001
Human–long-tailed macaque	0.91 (0.83–0.98)	53.423	*p* < 0.001
Human–leopard cat	0.42 (0.28–0.52)	56.468	*p* < 0.001
Human–Asian palm civet	0.10 (0.06–0.21)	101.98	*p* < 0.001
Human–small Indian civet	0.13 (0.06–0.27)	68.437	*p* < 0.001
Human–Javan ferret badger	0.15 (0.10–0.29)	43.072	*p* < 0.001
Human–Sunda porcupine	0.08 (0.04–0.17)	110.47	*p* < 0.001
Human–Malayan pangolin	0.14 (0.06–0.31)	33.49	*p* < 0.001

## Data Availability

The raw data are available upon reasonable request from the corresponding authors.

## Supplementary Materials

The following supporting information can be downloaded at https://www.mdpi.com/article/10.3390/ani13203217/s1: Figure S1: The presence of domestic dogs introduced by human activity within the confines of the Mount Merapi National Park region, (a) the domestic dog was brought by local people when looking for grass, and (b) the domestic dog was brought by poacher; Table S1: Covariate data for the modeling occupancy and detection probabilities; Table S2: Quantitative parameters of the level of interaction and co-occurrence between two species.
