# Supplementary material for: The Effects of Anthropogenic Disturbances on the Spatiotemporal Patterns of Medium–Large Mammals in Tropical Volcanic Landscapes
Source: Animals (Basel). 2023 Oct 14;13(20):3217. doi: 10.3390/ani13203217 (PMC10603758; doi:10.3390/ani13203217)
Supplement: Supplementary file 1 [file animals-13-03217-s001.zip › animals-2630233-supplementary.pdf]

Figure 1 the presence of domestic dogs introduced by human activity within the confines of the Gunung Merapi National Park region, (a) the domestic dog was brought by local people when looking for grass, and (b) the domestic dog was brought by poacher.

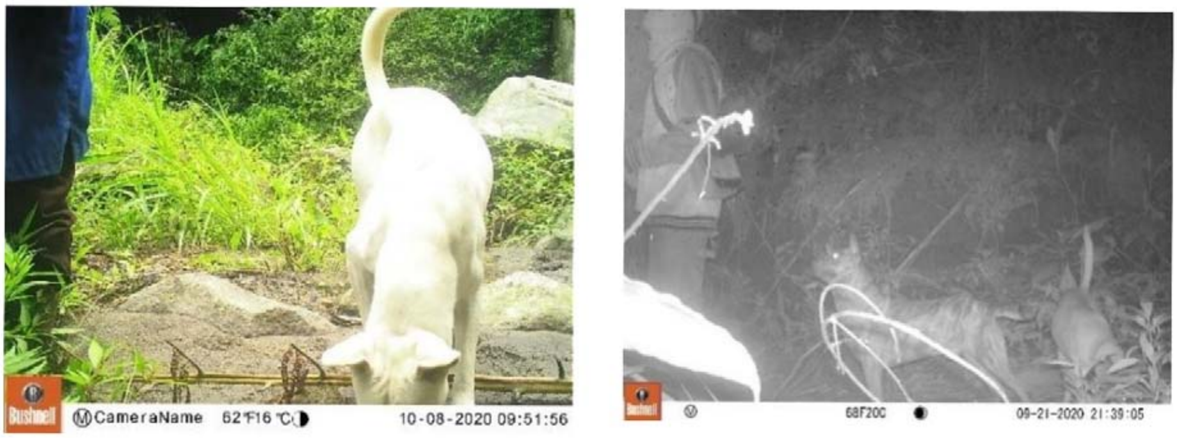

(a) (b)

Table S1 Covariate data for modeling occupancy and detection probabilities

Covariate	Symbol	Description
Height dense forest	HDF	Percentage of high-density forest within a radius of 100 m from the camera trap location (%)
Low dense forest	LDF	Percentage of low-density forest (%)
Slope	SLO	The slope value at the camera trap location (%)
Aspect	ASP	The quantitative value of the aspect at the camera trap location
Distance to settlement	DFS	The closest settlement distance to the camera trap (meter)
Distance to the mining area	DFM	The Euclidean distance of the sand and stone mining location to the camera trap (meter)
Distance to the top of a mountain	DFD	The euclidean distance of the lava dome to the camera trap (meter)
Distance to the grazing area	DFG	The closest flat distance of the grassy area to the camera trap (meter)
Altitude	HIG	The altitude level of the camera trap location (meter asl)

Distance 3 km to the top	DFP	The distance of the camera trap to the top of the mountain (meter)

Table S2 Quantitative parameters of the level of interaction and co-occurrence between two species

Parameter	Definition
ψA	Species A, probability of occupancy
ψBA	Species B, probability of occupancy when species A is absent
ψBa	Species B, probability of occupancy when species A is present
*p*A	Species A, probability of detection when species B is absent
*p*B	Species B, probability of detection when species A is absent
*r*A	Species A, probability of detection when species B is present
*r*BA	Species B, probability of detection with both species present and species A is detected during the sampling period
*r*Ba	Species B, probability of detection with both species present and species A is not detected during the sampling period
